# A New Hydroxylated Nonaprenylhydroquinone from the Mediterranean Marine Sponge *Sarcotragus spinosulus*

**DOI:** 10.3390/md9071210

**Published:** 2011-07-07

**Authors:** Charline Abed, Nathalie Legrave, Maeva Dufies, Guillaume Robert, Vincent Guérineau, Jean Vacelet, Patrick Auberger, Philippe Amade, Mohamed Mehiri

**Affiliations:** 1 Chemistry Laboratory of Biomolecules and Aroma, Nice Institute of Chemistry, UFR of Sciences, University of Nice-Sophia Antipolis, UMR-CNRS 6001, Parc Valrose, F-06108 Nice Cedex 02, France; E-Mails: charline.abed@univmed.fr (C.A.); nathalie.legrave@unice.fr (N.L.); philippe.amade@unice.fr (P.A.); 2 Marseille Oceanology Center, University of Aix-Marseille, CNRS UMR 6540 DIMAR, Endoume Marine Station, Batterie des Lions Street, 13007 Marseille, France; E-Mail: jean.vacelet@univmed.fr; 3 INSERM UMR 895, Team 2, Cell Death Differentiation and Cancer, Batiment ARCHIMED, 151 Saint-Antoine de Ginestiere Road, BP2 3194, 06204 Nice Cedex 3, France; E-Mails: dufies.maeva@etu.unice.fr (M.D.); guillaume.robert@unice.fr (G.R.); patrick.auberger@unice.fr (P.A.); 4 Centre de Recherche de Gif, Institut de Chimie des Substances Naturelles, CNRS, Avenue de la Terrasse, 91198 Gif-sur-Yvette Cedex, France; E-Mail: Vincent.Guerineau@icsn.cnrs-gif.fr

**Keywords:** sponges, *Sarcotragus spinosulus*, marine natural products, hydroxylated polyprenylhydroquinone, bioactivity

## Abstract

Chemical investigation of the Mediterranean sponge *Sarcotragus spinosulus* led to the isolation of a new hydroxylated nonaprenylhydroquinone, along with two known metabolites, hepta- and octaprenylhydroquinones. The structure of the new metabolite was assigned by extensive 1D and 2D NMR analyses and MS studies. The antileukemic effect of the three compounds towards the chronic myelogenous leukemia (CML) cells line K562 was also evaluated.

## 1. Introduction

Several linear and cyclic prenylated hydroquinones and related secondary metabolites have been isolated from sponges [1–6], tunicates [7,8], and algae [9,10]. A large number of linear polyprenylhydroquinones have been isolated from sponges, especially from the Irciniidae family (mainly from the genera *Ircinia* and *Sarcotragus*). Biological studies conducted on several polyprenylhydroquinones showed them to have a moderate antibacterial [11,12], antiviral [13], anti-inflammatory [14,15], and phospholipase A2 activity [15]. These linear polyprenylhydroquinones could be further divided in two main groups with polyprenylhydroquinones sulfates [16–20] and hydroxylated polyprenylhydroquinones. To date only four hydroxylated polyprenylhydroquinones [5,6,20,21] have been isolated from *Sarcotragus spinosulus* (under the name *Ircinia spinosula*). They have been shown to have an enhanced antibacterial, anti-inflammatory, cytotoxic [12,15], and antioxidant activity [22] potentially associated to the presence of the hydroxyl group on the prenyl side chain.

In the course of our search for bioactive marine natural products, we have investigated the Mediterranean marine sponge *Sarcotragus spinosulus* (Dictyoceratida, Irciniidae) collected from Callejones, Ceuta. In this paper we report the isolation and structural elucidation of a new hydroxylated nonaprenylhydroquinone, along with two known polyprenylhydroquinones, hepta- and octaprenylhydroquinones. We also report the antileukemic effect of the three compounds towards the chronic myelogenous leukemia (CML) cells line K562.

## 2. Results and Discussion

**Figure 1:**
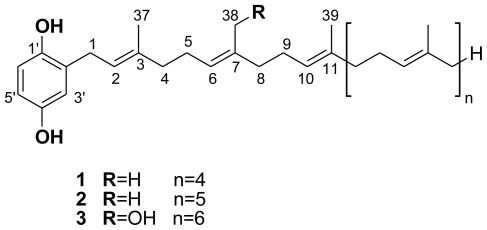
Structure of compounds **1**, **2**, and **3**.

The CH_2_Cl_2_/MeOH (1:1, v/v) crude extract of *Sarcotragus spinosulus* was fractionated by Flash Vacuum Liquid Chromatography, eluting with a gradient of decreasing polarity from H_2_O to MeOH. The subsequent MeOH fraction was purified by semipreparative reverse-phase HPLC (Phenomenex Luna C6-Pheny, 250 × 10 mm id, 5 μm, gradient H_2_O/MeCN/Formic Acid, 96:04:0.1 to 0:100:0.1) to afford pure compounds **1** (41.7 mg), **2** (30.6 mg), and **3** (4.7 mg) (Figure 1).

A preliminary NMR spectral analysis of all isolated compounds showed similarities and strongly supported the presence of a 2-polyprenylhydroquinone skeleton in each. Compounds **1**–**3** showed the same UV (280 nm) and IR (3350, 1500, 1445, 910, 785, 730 cm^−1^) spectra, indicating the presence of a monosubstituted hydroquinone structure. This was supported by the occurrence of three aromatic protons in the ^1^H NMR spectrum, two doublets at δ 6.56 (*J* = 8.5 Hz) and 6.52 (*J* = 3.0 Hz), and a double doublet at δ 6.57 (*J* = 8.5 and 3.0 Hz). An extensive examination of the spectroscopic data (IR, UV, 1D and 2D NMR, and MS spectra) of **1** and **2** led to their identification as hepta- and octaprenylhydroquinones, respectively. All spectral data of **1** and **2** were in agreement with previous published data [2,4].

The ^1^H- and ^13^C-NMR spectra of compound **3** were similar to those of compounds **1** and **2**, except for the presence of signals at δ 4.06 (2H) and δ 60.0, respectively, in the ^1^H and ^13^C NMR spectra (Table 1), assigned to the primary alcohol group of the side chain, and small differences in the chemical shifts around the OH group. Further examinations of the ^1^H- and ^13^C-NMR data of **3** led to its identification as a hydroxylated polyprenylhydroquinone, close analogues of which have been previously isolated from *S. spinosulus* [5,6,20,21]. The molecular formula C_51_H_78_O_3_ of **3** was deduced from the HR-MALDITOF-MS data which showed a pseudomolecular adduct ion at *m/z* 845.4874 [M + Ag]^+^ (Calcd. for C_51_H_78_^107^AgO_3_ 845.5001) [23]. Thus, NMR and MS data of **3** led to its identification as a hydroxylated nonaprenylhydroquinone in which one of the methyls has been oxidized to hydroxymethylene. The position of the OH group was unequivocally assigned on the second isoprene unit based on the shift of the olefinic proton H-6 to δ 5.25 (*vs*. 5.10 in polyprenylhydroquinone), and key H-4/H-5/H-6 COSY correlations. Confirmation was given by the key H-38/C-6, H-38/C-7, and H-38/C-8 HMBC correlations. Thus, the new natural product **3** was identified as 2′-[38-Hydroxy]nonaprenyl-1′,4′-hydroquinone. Finally, the configurations of double bonds were all assigned as *E* by ^1^H and ^13^C NMR chemical shifts (δ 1.68–1.54 and 17.8–16.1 for ^1^H and ^13^C, respectively) of the vinyl methyls [24].

To the best of our knowledge this is the first report on the isolation and structure identification of a hydroxylated nonaprenylhydroquinone. Until now, four hydroxylated polyprenylhydroquinones have been reported: two heptaprenyl bearing the OH group on the first [20] and fifth prenyl moiety [6]; and two octaprenyl congeners bearing the OH group on the fourth [21] and fifth [5] isoprene unit.

From a chemotaxonomic point of view hydroxylated polyprenylhydroquinones could constitute potential markers for *S. spinosulus* species.

The metabolites **1**–**3** were evaluated for their potential antileukemic effect towards the chronic myelogenous leukemia (CML) cells line K562, which is widely used for cytotoxicity assays. The effect of compounds **1**–**3** was compared with that of Imatinib, the leading compound to treat patients suffering CML. This compound has proven very efficient in killing Bcr-Abl-positive cells in a caspase-dependent manner [25,26]. The IC_50_ values for Imatinib and compounds **1**–**3** for loss of cell metabolism (XTT assay) and cell number are given in Table 2.

Compounds **1** and **2** inhibited cell metabolism and cell number with very similar IC_50_ values (around 10 μM). Compound **3** was less efficient that the two former compounds with IC_50_ values of 193 and 191 μM, respectively. As a control, Imatinib was shown to inhibit cell metabolism and cell number with IC_50_ values of 0.4 and 0.5 μM, respectively, in agreement with previous results [26]. As compounds **1** and **2** were also found to induce annexin V externalisation in K562 cells (not shown), it is likely that the main mechanism by which both compounds inhibit cells metabolism and cells number is by inducing apoptosis.

## 3. Experimental Section

### 3.1. General

All organic solvents used for material extraction were of analytical grade and purchased from Merck (Darmstadt, Germany). Acetonitrile used for HPLC was of HPLC-grade and purchased from Fisher, USA. Formic acid of HPLC grade was purchased from Acros, USA. 2,5-Dihydroxybenzoic acid (DHB, used as the matrix for MALDI-TOF experiments, was of the highest grade available and used without further purification) and Silver trifluoroacetate (AgTFA, used as the cationizing agent) were purchased from Sigma Aldrich Co. The Chromabond C18 preparative column used for flash chromatography was obtained from Merck, USA. Imatinib was kindly provided by Novartis Pharma. UV measurements were performed on a Varian Cary 300 Scan UV-visible spectrometer. IR spectra were obtained with a Perkin-Elmer Paragon 1000 FT-IR spectrometer. Flash chromatography was performed on an Armen Instrument Spot Liquid Chromatography system, the detection wavelength was set at 254 nm. HPLC purifications were carried out on a Waters 600 system equipped with a Waters 717 plus autosampler, a Waters 996 photodiode array detector, and a Sedex 55 evaporative light-scattering detector (SEDERE, France). Detection wavelengths were set at 214, 254 and 280 nm. ^1^H and ^13^C NMR spectra were recorded with 500 MHz Bruker Avance NMR spectrometers. Chemical shifts (δ) are recorded in ppm with CD_3_OD (δ_H_ 3.31 ppm and δ_C_ 49.0 ppm) as internal standards with multiplicity (s, singlet; d, doublet; t, triplet; m, multiplet). High resolution mass spectra (HRMALDITOFMS) were conducted on a Perseptive Voyager DE-STR MALDI-TOF mass spectrometer (Perseptive Biosystems, Framingham, MA, USA), equipped with a 337 nm pulsed nitrogen laser (20 Hz) and a Acqiris^®^ 2 GHz digitizer board, was used for all experiments. Mass spectra were obtained in reflectron positive ion mode with the following settings: accelerating voltage 20 kV, grid voltage 62% of accelerating voltage, extraction delay time of 100 ns. The laser intensity was set just above the ion generation threshold to obtain peaks with the highest possible signal-to-noise (S/N) ratio without significant peak broadening. All data were processed using the Data Explorer software package (Applied Biosystems).

### 3.2. Sponge Material

**Figure 2:**
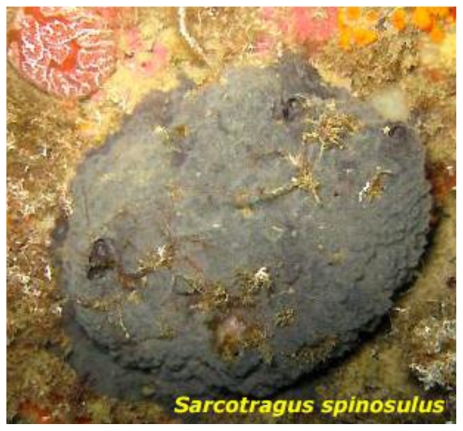
*Sarcotragus spinosulus*.

A sponge sample of *Sarcotragus spinosulus* (Schmidt, 1862) (Figure 2) (Demospongiae, Dictyoceratida, Irciniidae) was collected by hand using scuba at a depth of about 10 m from Callejones, Ceuta in June 2009. Taxonomic examination of the sponge was made by the authors (C.A. and J.V). The sponge shape is massive and subspherical. The color *in situ* is dark grey exterior, white to beige interior, and in EtOH the flesh color turned slightly to reddish. The texture is difficult to tear and has a firm and compressible consistency. The ectosome is unarmoured, thick with conules (1 to 2 mm height) irregularly scattered over the entire surface. The choanosome is cavernous. Its skeleton consists of laminated primary and secondary fibers and comprises very dense spongin filaments. Primary fibers (90 to 180 μm diameter) are pithed and clear of foreign detritus. Secondary fibers (50 to 100 μm diameter) are uncored, and the spongin filaments are long with a very thin diameter of 0.7 to 2 μm. A sponge sample (090618Ce7-05) is kept in the sponge collection of the Centre d’Océanologie de Marseille (Station Marine d’Endoume, France). The sponge was kept frozen until the extraction process.

### 3.3. Extraction and Isolation

A portion of *S. spinosulus* was freeze-dried and ground to obtain a dry powder (20 g), which was exhaustively extracted with a mixture of MeOH/CH_2_Cl_2_ (1:1, v/v) to yield 2.2 g of the crude extract after concentration under reduced pressure. The crude extract was fractionated by RP-C18 flash chromatography (elution with a decreasing polarity gradient of H_2_O/MeOH from 1:0 to 0:1, then MeOH/CH_2_Cl_2_ from 1:0 to 0:1) (flow: 10 mL/min). The MeOH fraction (448 mg) was then subjected to semi-preparative HPLC-DAD (Phenomenex Luna C6-Pheny, 250 × 10 mm id, 5 μm) with a gradient of H_2_O/MeCN/Formic acid 96:4:0.1 to 0:100:0.1) (flow: 3.0 mL/min, injection volume: 100 μL) to afford pure compounds **1**–**3**. All of them were identified by a combination of spectroscopic methods (1D and 2D NMR, MS) and comparison with the literature data [2,4].

### 3.4. Characterization Data

Compound **1**: HR-MALDITOF-MS *m/z* 693.3808 [M + Ag]^+^ (Calcd. for C_41_H_62_^107^AgO_2_, 693.3795); For ^1^H NMR and ^13^C NMR data see [2,4].

Compound **2**: HR-MALDITOF-MS *m/z* 761.4389 [M + Ag]^+^ (Calcd. for C_46_H_70_^107^AgO_2_, 761.4421); For ^1^H and ^13^C NMR data see [2,4].

Compound **3**: yellow solid; IR (CHC1_3_) 3350, 1500, 1445, 910, 785, 730 cm^−1^, UV λ_max_ (MeOH) 280 nm (ɛ 2500); HR-MALDITOF-MS *m/z* 845.4874 [M + Ag]^+^ (Calcd. for C_51_H_78_^107^AgO_3_, 845.5001); For ^1^H NMR (500 MHz) and ^13^C NMR (125 MHz) data see Table 1.

### 3.5. Biological Activity

Cell Line: The human CML K562 cell line was provided by ATCC and were grown at 37 °C under 10% CO_2_ in RPMI 1640 medium (Gibco BRL, Paisley, UK) supplemented with 5% FCS (Gibco BRL, Paisley, UK) completed with 50 units/mL penicillin, 50 mg/mL streptomycin and 1 mM sodium pyruvate.

Measurement of Cell Metabolism (XTT): Cells (15 × 10^4^/mL) were incubated with **1**, **2** or **3** for the times indicated. 50 mL of XTT reagent (sodium 39-[1-(phenylaminocarbonyl)-3,4-tetrazolium]-bis (4-methoxy-6-nitro)benzene sulfonic acid hydrate) was added to each well. Absorbance of the formazan dye produced by metabolically active cells was measured at 490 nm as described earlier [27]. Each assay was performed in quadruplicate.

Cell death measurement: After the indicated treatment, cells were harvested and percentage of viability was measured by propidium iodide (PI) staining (0.5 Ag/mL) and flow cytometry analysis in FL-3.

## 4. Conclusions

A new hydroxylated nonaprenylhydroquinone, 2′-[38-Hydroxy]nonaprenyl-1′,4′-hydroquinone, along with two known metabolites, hepta- and octaprenyl-1′,4′-hydroquinones, have been isolated from the Mediterranean marine sponge *Sarcotragus spinosulus*. These compounds exhibited a good activity against K562 cells which will warrant further analysis at the molecular level and offer promising opportunities for the development of new antitumor agents.

## Figures and Tables

**Table 1:** ^1^H NMR (500 MHz, CD_3_OD) and ^13^C NMR (125 MHz, CD_3_OD) data of compound **3**.

No.	δ_C_ (ppm)	Mult.	δ_H_ (ppm)	*J* (Hz)	Mult.	COSY	HMBC
1′	148.9	qC					
2′	130.2	qC					
3′	117.2	CH	6.52	3.00	d	1	1, 1′, 4′, 5′
4′	151.1	qC					
5′	113.8	CH	6.41	8.50, 3.00	dd	6′	3′, 4′
6′	116.5	CH	6.56	8.50	d	5′	1, 1′, 2, 5′
1	29.1	CH_2_	3.22	7.33	d	2	1′, 2′, 3, 3′
2	124.0	CH	5.30		m	1	1, 4
3	136.8	qC					
4	40.9	CH_2_	1.98		m	5	
5	27.7	CH_2_	2.16		m	4, 6	3, 4, 6, 7
6	128.8	CH	5.25		m	5	
7	139.4	qC					
8	35.9	CH_2_	2.11		m	9	9, 6, 10, 38
9	27.7	CH_2_	2.05		m	8, 10	
10	125.6	CH	5.10		m	9	
11	135.8	qC					
12	40.9	CH_2_	1.96		m		
13	27.7	CH_2_	2.05		m		
14	125.6	CH	5.10		m		
15	135.8	qC					
16	40.9	CH_2_	1.96		m		
17	27.7	CH_2_	2.05		m		
18	125.6	CH	5.10		m		
19	135.8	qC					
20	40.9	CH_2_	1.96		m		
21	27.7	CH_2_	2.05		m		
22	125.6	CH	5.10		m		
23	135.8	qC					
24	40.9	CH_2_	1.96		m		
25	27.7	CH_2_	2.05		m		
26	125.6	CH	5.10		m		
27	135.8	qC					
28	40.9	CH_2_	1.96		m		
29	27.7	CH_2_	2.05		m		
30	125.6	CH	5.10		m		
31	135.8	qC					
32	40.9	CH_2_	1.96		m		
33	27.7	CH_2_	2.05		m		
34	125.6	CH	5.10		m		
35	135.8	qC					
36	25.9	CH_3_	1.65		s		34,45
37	16.2	CH_3_	1.69		s	1,2	2,3,4
38	60.0	CH_2_	4.06		s		6,7,8
39	16.2	CH_3_	1.58		s		
40	16.2	CH_3_	1.58		s		
41	16.2	CH_3_	1.58		s		
42	16.2	CH_3_	1.58		s		
43	16.2	CH_3_	1.58		s		
44	16.2	CH_3_	1.58		s		
45	17.8	CH_3_	1.58		s		

**Table 2:** IC_50_ values for Imatinib and compounds **1**–**3** for loss of cell metabolism (XTT assay) and cell number. Cells (15 × 10^4^/mL) were incubated for 48 h at 37 °C with either increasing concentration of compounds **1**–**3** in the 2.5–250 μM range or with Imatinib in the 0.1–2.5 μM range. Cell metabolism was measured using the XTT assay and cell numbers was assessed by flow cytometry as indicated in the Experimental section. IC_50_ values are representative of three experiments made in quadruplicate.

Compounds	Cell metabolism (IC_50_)	Cell number (IC_50_)
**1**	8	7
**2**	10	12
**3**	193	191
Imatinib	0.4	0.5
